# Risk, Precipitating, and Perpetuating Factors in Functional Neurological Disorder: A Systematic Review Across Clinical Subtypes

**DOI:** 10.3390/brainsci15090907

**Published:** 2025-08-23

**Authors:** Ioannis Mavroudis, Katerina Franekova, Foivos Petridis, Alin Ciobîca, Gabriel Dăscălescu, Emil Anton, Ciprian Ilea, Sotirios Papagiannopoulos, Dimitrios Kazis

**Affiliations:** 1 Department of Neuroscience, Leeds Teaching Hospital, NHS Trust Leeds, Leeds LS17 7HY, UK; ioannis.mavroudis@gmail.com; 2 Institute of Health Sciences, University of Leeds, Leeds LS2 9JT, UK; katarina.franekova@qcentrum.sk; 3 Third Department of Neurology, Aristotle University of Thessaloniki, 541 24 Thessaloniki, Greece; f_petridis83@yahoo.gr (F.P.); spapagia@auth.gr (S.P.); dimitrios.kazis@gmail.com (D.K.); 4 Academy of Romanian Scientists, 050085 Bucharest, Romania; 5 Department of Biology, Faculty of Biology, “Alexandru Ioan Cuza” University of Iasi, 700506 Iasi, Romania; alin.ciobica@uaic.ro (A.C.); gabidascalescu2001@gmail.com (G.D.); 6 CENEMED Platform for Interdisciplinary Research, University of Medicine and Pharmacy “Grigore T. Popa”, 700115 Iasi, Romania; 7 “Olga Necrasov” Center, Department of Biomedical Research, Romanian Academy, 010071 Iasi, Romania; 8 “Ioan Haulica” Institute, Apollonia University, 700511 Iasi, Romania; 9 Faculty of Medicine, University of Medicine and Pharmacy “Grigore T. Popa”, 700115 Iasi, Romania; cilea1979@yahoo.com

**Keywords:** Functional Neurological Disorder, risk factors, Psychogenic Nonepileptic Seizures, functional cognitive disorder, biopsychosocial model, systematic review

## Abstract

***Background:*** Functional Neurological Disorder (FND) encompasses conditions with neurological symptoms inconsistent with structural pathology, arising instead from complex interactions between psychological, biological, and social factors. Despite growing research, the etiological and risk factor landscape remains only partially understood, complicating diagnosis and treatment. ***Objective:*** This systematic review maps risk factors for major FND subtypes such as functional seizures (psychogenic non-epileptic seizures or PNES), functional cognitive disorder (FCD), functional movement disorders (FMD), functional weakness and sensory disturbances, functional visual symptoms, and functional gait abnormalities by categorizing predisposing, precipitating, and perpetuating influences. ***Methods:*** A systematic search of PubMed, PsycINFO, Scopus, and Web of Science initially identified 245 records. After removal of 64 duplicates, 181 studies were screened by title and abstract. Of these, 96 full texts were examined in detail, and finally 23 studies met the predefined inclusion criteria. Data were extracted and analyzed thematically within a biopsychosocial framework, with results summarized in subtype-specific profiles. ***Results:*** Childhood adversity, especially emotional, physical, or sexual abuse, emerged as a robust and consistent predisposing factor across PNES cohorts. Psychiatric history (notably anxiety, depression, and PTSD), neurodevelopmental traits (more frequent in FCD), and personality patterns such as alexithymia and somatization also contributed to vulnerability. Precipitating influences included acute psychological stress, intrapersonal conflict, or concurrent medical illness. Perpetuating factors comprise maladaptive illness beliefs, avoidance behaviors, insufficient explanation or validation by healthcare providers, and secondary gains related to disability. While several risk factors were shared across subtypes, others appeared subtype-specific (trauma was especially associated with PNES, whereas neurodevelopmental traits were more characteristic of FCD). ***Conclusions:*** FND arises from a dynamic interplay of predisposing, precipitating, and perpetuating factors, with both shared and subtype-specific influences. Recognizing this heterogeneity can enhance diagnostic precision, guide tailored intervention, and inform future research into the neurobiological and psychosocial mechanisms underlying FND.

## 1. Introduction

Functional Neurological Disorder (FND) represents a prevalent yet historically misunderstood condition situated at the intersection of neurology and psychiatry. It is defined by neurological symptoms (such as seizures, motor disturbances, cognitive complaints, or gait abnormalities) that are incongruent with recognized structural or pathophysiological mechanisms [1]. Once viewed primarily as a diagnosis of exclusion, FND is now understood as a positive clinical entity, identified through specific examination signs and characteristic patterns of symptom expression [2]. This reconceptualization has significantly improved diagnostic confidence, although therapeutic uncertainty and stigma remain pervasive. FND poses a substantial burden on both patients and healthcare systems. Prevalence estimates suggest that it accounts for approximately 4–12% of referrals to neurology outpatient clinics, with even higher rates reported in epilepsy monitoring units [3,4]. These figures highlight the need for increased diagnostic clarity and more effective, individualized treatment approaches. In recent decades, mounting empirical evidence has supported a biopsychosocial model of FND, emphasizing the dynamic interactions between biological predisposition, psychosocial stressors, and maladaptive cognitive-behavioral patterns in the development and maintenance of functional symptoms [5,6]. This framework marks a departure from earlier, reductionist models centered on hysteria, malingering, or primary gain, instead promoting a compassionate and neurobiologically plausible understanding of patient experience [7,8].

### 1.1. From Description to Mechanisms: The Emergence of Risk Profiling

The shift from descriptive diagnosis to mechanistic understanding has redirected attention toward the complex architecture of risk factors (those predisposing, precipitating, and perpetuating elements that influence the onset, persistence, and recurrence of FND) [9]. Clarifying these factors is essential for transitioning from reactive management to proactive and personalized care. A risk-informed approach allows clinicians to better identify vulnerable individuals, understand symptom trajectories, and tailor interventions accordingly.

Multiple landmark studies have begun to chart this terrain, revealing a rich but fragmented body of evidence. Central to this evolving landscape is the need to synthesize these findings into a unified, subtype-sensitive framework that reflects the heterogeneity of FND.

### 1.2. Predisposing Factors: A Foundation of Vulnerability

Robust evidence implicates early-life adversity, particularly childhood trauma (emotional, physical, or sexual), as a prominent predisposing factor. Studies by Roelofs et al. [10] and Aybek et al. [9] reported significantly elevated trauma exposure in patients with FND (most notably in those with psychogenic non-epileptic seizures—PNES). Neuroimaging data suggest that such trauma may result in long-term alterations in limbic-prefrontal connectivity, with downstream effects on emotion regulation and motor control.

Attachment insecurity and impaired affect regulation have also been identified as vulnerability markers. Ludwig et al. [6] found that individuals with FND often demonstrate fearful or dismissive attachment styles, which may hinder resilience and disrupt trust in medical relationships. Neurodevelopmental features, including autistic traits, ADHD symptoms, and altered sensory processing, are particularly relevant in functional cognitive disorder (FCD), as shown by Demartini et al. [11] and Silverberg et al. [12]. These traits may interfere with attentional control, metacognition, and predictive coding, thereby shaping symptom perception.

Psychiatric comorbidities, particularly mood and anxiety disorders, are frequently observed in FND cohorts [2,13]. However, a substantial subset of patients (up to one-third) present without any diagnosable psychiatric condition [1], underscoring the non-obligatory role of psychopathology. Personality traits may further modulate vulnerability. Cloninger’s biosocial model [14] and findings by Binzer et al. [15] suggest that temperamental patterns, such as high harm avoidance or obsessive-compulsive tendencies, interact with environmental stressors in shaping risk. Family history and modeling, while less extensively studied, appear relevant. Cabreira et al. [16] observed higher rates of functional symptoms among first-degree relatives, suggesting combined genetic and psychosocial transmission.

### 1.3. Precipitating Factors: Triggers of Onset

Functional symptoms often emerge in the aftermath of identifiable stressors or somatic events. Physical illness (e.g., injury, infections, or pain), psychological trauma (e.g., bereavement or interpersonal conflict), and invasive medical procedures may all act as precipitants. Stone et al. reported that over 60% of patients with functional weakness or sensory symptoms recalled a recent acute trigger [4]. In FCD, stressors such as sleep deprivation, illness, and occupational pressure are frequently implicated [13]. In PNES, unresolved trauma and relational stressors, especially in the context of limited emotional regulation capacity, are common antecedents [6,17].

Importantly, iatrogenic factors, including ambiguous diagnostic labeling, unnecessary investigations, or perceived invalidation, may themselves constitute triggers. The experience of medical uncertainty or misdiagnosis can amplify somatic focus, erode trust, and contribute to symptom escalation [8,18].

### 1.4. Perpetuating Factors: Mechanisms of Maintenance

FND is frequently characterized by a relapsing or persistent course, often maintained by maladaptive beliefs and behavioral responses. These include somatic hypervigilance, catastrophic misinterpretations of bodily sensations, functional avoidance, and reinforcement through secondary gains (e.g., social attention, disability benefits) [5,18]. The therapeutic relationship plays a pivotal role: poor communication, lack of explanation, or clinician skepticism may exacerbate patient anxiety and diagnostic confusion. Studies by Sharpe et al. and Carson et al. emphasized how clinician behavior can either support recovery or, conversely, entrench illness behavior diagnostic uncertainty [8,19].

### 1.5. Subtype-Specific Risk Patterns

While many mechanisms are shared across FND subtypes, certain patterns appear to be subtype-specific. PNES is more strongly associated with trauma and dissociation [7,10], whereas FCD is linked to attentional dysregulation and neurodevelopmental traits [20]. Functional gait disturbances and visual symptoms, as shown by Geroin et al. and Lim et al., often involve heightened interoceptive sensitivity and environmental reactivity [21,22]. These distinctions underscore the need for differential risk mapping within the broader FND construct.

### 1.6. Rationale for the Present Review

Although the literature on FND risk factors is expanding, it remains fragmented, with most studies focusing on isolated variables in narrowly defined populations. To date, no review has comprehensively synthesized risk factor data across all major FND subtypes using a systematic framework. The current review seeks to address this gap by providing an integrated, evidence-based map of predisposing, precipitating, and perpetuating influences in FND.

By aligning findings within a structured biopsychosocial model and highlighting subtype-specific distinctions, this synthesis aims to inform clinical formulation, guide individualized intervention, and support ongoing mechanistic research. Most importantly, it advocates for a paradigm in which functional symptoms are recognized not as inexplicable or fabricated, but as meaningful expressions of complex biopsychosocial processes, processes that, when understood, can be compassionately and effectively addressed.

## 2. Materials and Methods

### 2.1. Study Design

This study employed a systematic review and narrative synthesis methodology, aiming to identify, extract, and integrate empirical evidence regarding predisposing, precipitating, and perpetuating factors implicated in Functional Neurological Disorders (FNDs). The review encompassed all major clinical subtypes, including functional seizures (PNES), functional movement disorders (FMD), functional cognitive disorder (FCD), functional weakness and sensory symptoms, functional gait disturbances, and functional visual symptoms.

The protocol adhered to established methodological guidance for systematic reviews and was conducted in accordance with the PRISMA 2020 (Preferred Reporting Items for Systematic Reviews and Meta-Analyses) framework to ensure methodological transparency and reproducibility.

### 2.2. Eligibility Criteria

Studies were included based on the following predefined inclusion criteria:

- Population: adults and/or adolescents diagnosed with FND or any of its recognized subtypes (PNES, FMD, FCD, functional weakness/sensory symptoms, functional gait, or visual manifestations).
- Study design: original empirical research employing quantitative (cross-sectional, cohort, or case–control), qualitative, or mixed-methods design.
- Focus: studies that explicitly assessed or discussed risk factors, including those categorized as predisposing (e.g., early-life adversity, psychiatric history), precipitating (e.g., acute stressors, somatic illness), or perpetuating (e.g., cognitive, behavioral, or social maintenance mechanisms).
- Language: publications written in English.
- Publication type: peer-reviewed journal articles, scholarly book chapters, or authoritative theoretical sources with original content.

Exclusion criteria:

- Studies focusing solely on treatment efficacy without discussion of risk factors.
- Research involving non-human subjects.
- Narrative or systematic reviews without primary data.
- Publications lacking sufficient methodological or contextual detail for meaningful data extraction.

### 2.3. Search Strategy

A comprehensive literature search was conducted across four major databases: PubMed, PsycINFO, Scopus, and Web of Science. The search strategy combined Medical Subject Headings (MeSH) and keyword terms using Boolean operators, targeting the intersection between FND subtypes and risk-related constructs. Search key included: “Functional Neurological Disorder” OR “ Conversion Disorder” OR “Functional Seizures” “Psychogenic Nonepileptic Seizures” OR “Non-Epileptic Attack Disorder” OR “Dissociative Convulsions” OR “Dissociative Motor Disorder” OR “Functional Movement Disorder” AND “Risk Factors” OR “Predisposing” OR “Precipitating” OR “Perpetuating” Or “Etiology” OR “ Psychosocial Factors” OR “Trauma” OR “Comorbidity”.

As an example, the PubMed search string was as follows:

(“functional neurological disorder” [tiab] OR “conversion disorder” [tiab] OR “psychogenic nonepileptic” [tiab] OR “non-epileptic attack disorder” [tiab] OR “dissociative convulsions” [tiab] OR “dissociative motor disorder”) AND (“risk factor” [tiab] OR “predisposing” [tiab] OR “precipitating” [tiab] OR “perpetuating” [tiab] OR “prognosis” [tiab] OR “treatment response” [tiab]), where [tiab] in a PubMed query means that the search is limited to the title and abstract fields of the indexed articles.

The search was limited to publications indexed from database inception through May 2025. In addition, backward citation tracking and hand-searching of relevant reviews were performed to capture supplementary sources. All references were manually organized and tracked using structured spreadsheets to document duplicate removal, screening decisions, and reasons for exclusion, in accordance with the PRISMA guidelines.

### 2.4. Study Selection Process

**Figure 1:**
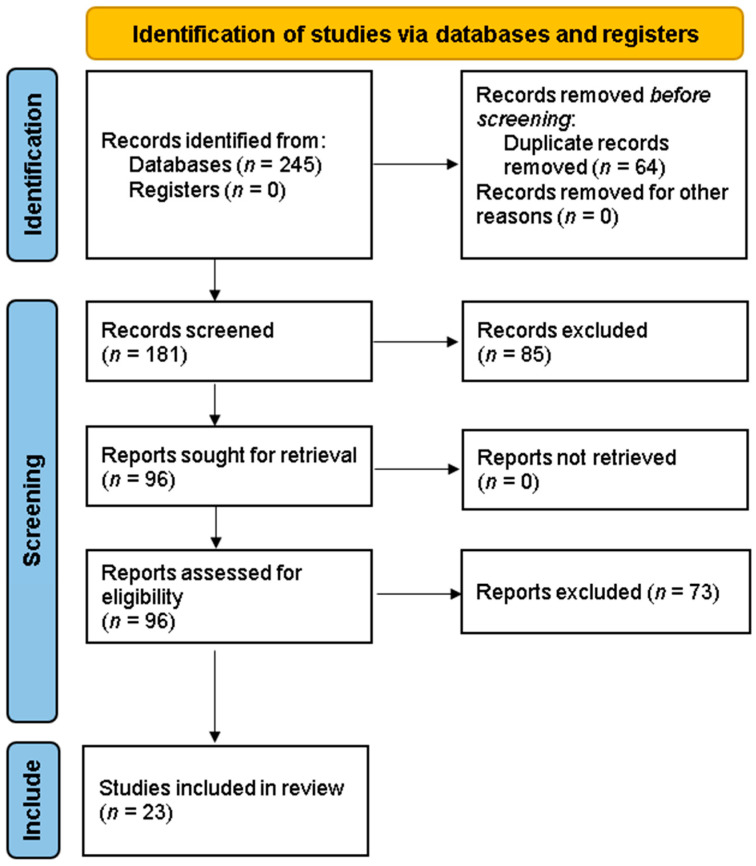
PRISMA 2020 flow diagram. Flowchart depicting the identification, screening, eligibility, and inclusion process of studies used in the systematic review (*n* = 23 included).

A total of 245 records were identified through database searching. After removal of 64 duplicates, 181 unique studies remained for screening. Two independent reviewers evaluated titles and abstracts against the eligibility criteria, resulting in 96 full-text articles being retrieved for detailed assessment. Discrepancies at any stage were resolved through discussion or, when necessary, adjudication by a third reviewer. Ultimately, 23 studies met the inclusion criteria and were included in the final synthesis. The study selection process is detailed in the PRISMA flowchart (Figure 1).

This systematic review was not registered in PROSPERO or any similar database, as the review process had already been initiated and substantially progressed by the time registration was considered. To maintain transparency, we adhered closely to the PRISMA guidelines in study identification, screening, and reporting.

### 2.5. Data Extraction

Data from the included studies were extracted using a standardized, pre-piloted template. The following variables were systematically recorded:

- Author(s), year of publication, and study title;
- FND subtype(s) examined;
- Study design and sample characteristics;
- Type(s) of risk factor (predisposing, precipitating, perpetuating);
- Specific factors identified and their operational definitions;
- Method of assessment (e.g., clinical interview, diagnostic criteria, validated instruments);
- Key findings, including statistical outcomes where applicable;
- Source quality indicators and level of evidence.

Two authors independently reviewed all extractions to ensure accuracy and completeness.

### 2.6. Risk of Bias and Quality Assessment

Each study was assessed for methodological quality using tools appropriate to its design:

- Observational studies were appraised using the Newcastle–Ottawa Scale (NOS), evaluating selection, comparability, and outcome domains.
- Qualitative studies were evaluated using the Critical Appraisal Skills Program (CASP) checklist, focusing on research design, data validity, and reflexivity.

**Figure 2:**
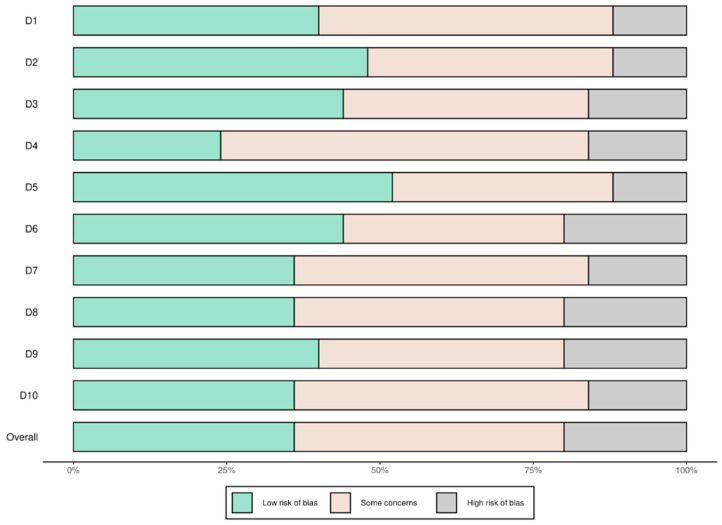
**Risk of bias summary.** Proportional stacked bar plot summarizing the risk of bias across all domains using standardized assessment tools. Low-, moderate-, and high-risk classifications are color-coded.

For both tools, risk of bias was categorized as low, moderate, or high, according to predefined scoring thresholds. Figure 2 illustrates the distribution of risk across ten methodological domains, including participant selection, comparability, outcome assessment, sampling strategy, data collection, and analytical rigor.

Two reviewers independently rated each study. Disagreements were addressed through consensus or resolved by a third-party reviewer. Quality ratings were used to contextualize findings but not to exclude studies.

References for the PRISMA 2020 guidelines [22], the Newcastle–Ottawa Scale (NOS) [23], and the Critical Appraisal Skills Program (CASP) [24] checklist were cited for transparency.

### 2.7. Data Synthesis and Analysis

A thematic synthesis approach was applied to organize the extracted findings into the three conceptual domains:

- Predisposing factors (e.g., developmental, genetic, psychiatric, or personality-related vulnerabilities);
- Precipitating factors (e.g., recent stressors, illness, interpersonal conflict, or medical procedures);
- Perpetuating factors (e.g., illness beliefs, maladaptive behaviors, or clinician interactions);
- Findings were stratified by FND subtype, enabling comparison across functional seizures, cognitive disorder, movement disorder, and other presentations. Extracted data were tabulated to allow pattern identification across domains and subtypes.

Finally, a narrative synthesis was conducted to integrate thematic patterns, highlight areas of convergence and divergence, and discuss implications for clinical formulation, intervention development, and future research.

## 3. Results

A total of 245 records were retrieved through the database search. After removal of duplicates and initial screening, 96 full-text articles were assessed for eligibility. Following detailed evaluation based on the predefined inclusion and exclusion criteria, 23 studies were deemed suitable for inclusion in this systematic review. The study selection process is outlined in Figure 1 (PRISMA Flow Diagram).

Although the authors adhered to established best practices in conducting systematic reviews and followed the PRISMA 2020 statement [22], the protocol for this review was not registered with PROSPERO. This decision was made as the study had already progressed beyond the protocol development stage by the time PROSPERO registration was considered, and retrospective registration was not permitted under its policy guidelines. Nonetheless, all methods and procedures were transparently documented in advance and followed consistently throughout the review process.

Included studies were systematically synthesized using a biopsychosocial framework. Risk factors were categorized into three primary domains: predisposing factors (long-term vulnerabilities), precipitating factors (acute triggers), and perpetuating factors (mechanisms maintaining symptoms). These domains were examined across six core FND subtypes: psychogenic non-epileptic seizures (PNES), functional cognitive disorder (FCD), functional movement disorder (FMD), functional weakness and sensory symptoms, functional visual symptoms, and functional gait disorder.

The characteristics of the studies included in this manuscript are presented in Table 1.

Methodological quality and risk of bias for the included studies were assessed using standardized tools summarized in Figure 2.

In Figure 2, the ten domains reflect the criteria applied from the Newcastle–Ottawa Scale (for observational studies) and the CASP checklist (for qualitative studies): selection, comparability, outcome assessment, sampling strategy, data collection, data analysis, reporting clarity, ethical consideration, reflexivity, and overall rigor.

### 3.1. Psychogenic Non-Epileptic Seizures (PNES)

#### 3.1.1. Predisposing Factors

A substantial body of evidence indicates that early-life trauma, including sexual, physical, and emotional abuse, constitutes a major predisposing factor for PNES [7,10]. Neuroimaging findings support this association, revealing altered limbic-prefrontal connectivity in trauma-exposed patients, suggesting that maladaptive memory encoding and emotional dysregulation may disrupt motor control circuits [10]. Psychiatric comorbidities, particularly anxiety and depressive disorders, were observed in over two-thirds of patients, frequently preceding the onset of functional seizures [1,2,3,6]. Personality profiles, including traits of harm avoidance, emotional lability, and suggestibility, as well as elevated Cluster B and Cluster C features (notably histrionic and obsessive-compulsive traits), have also been implicated [11,12]. Additionally, familial modeling and intergenerational transmission of illness behavior were reported, indicating that symptom expression may be learned within emotionally enmeshed family environments [13]. The association between childhood trauma and PNES was reported in over two-thirds of included studies, reflecting a strong and consistent relationship across methodologies.

#### 3.1.2. Precipitating Factors

Acute psychosocial stressors, such as bereavement, divorce, financial hardship, or interpersonal conflict, were frequently reported preceding symptom onset. More than half of PNES patients identified a recent adverse life event [4,6,17]. Somatic triggers, including infections, head trauma and epileptic seizures, may initiate maladaptive bodily monitoring and threat interpretation, thereby precipitating episodes [4,19].

#### 3.1.3. Perpetuating Factors

Symptom chronicity was strongly associated with catastrophic illness beliefs, particularly perceptions of functional episodes as life-threatening or uncontrollable [5]. Reinforcement mechanisms (social attention, caregiver over-accommodation, and disability compensation) contributed to the maintenance of symptoms [18]. Iatrogenic influences, including ambiguous diagnostic communication, clinician skepticism, and prolonged diagnostic delays, were also implicated in perpetuating illness behavior [8].

Table 2 summarizes the key predisposing, precipitating, and perpetuating factors as PNES as reported in the included studies.

### 3.2. Functional Cognitive Disorder (FCD)

#### 3.2.1. Predisposing Factors

Neurodevelopmental traits, particularly autism spectrum characteristics and attention-deficit/hyperactivity symptoms, were overrepresented in FCD populations [14,15,20]. Anxiety and depressive disorders were prevalent, occurring in over 70% of cases [13,20]. Personality features, including perfectionism, cognitive rigidity, and obsessive-compulsive tendencies, may predispose individuals to interpret normal cognitive lapses as pathological [11,12].

Neurodevelopmental traits were less universally reported but were consistently replicated in at least three independent cohorts, suggesting a moderate level of evidence.

#### 3.2.2. Precipitating Factors

More than half of participants reported recent stressors or psychological disruptions, such as infections, chronic fatigue, sleep disturbances, and occupational burnout [6,13,19], likely increasing vulnerability to misinterpretations of attentional lapses or memory errors.

#### 3.2.3. Perpetuating Factors

Persistent symptoms were associated with dysfunctional illness models, particularly fear of early-onset dementia or neurodegeneration despite normal testing results [5,20]. Excessive medical investigations without adequate feedback, coupled with poor clinician-patient communication, reinforced maladaptive beliefs and heightened distress [8].

Table 3 presents a synthesis of the main predisposing, precipitating, and perpetuating factors identified in FCD.

### 3.3. Functional Movement Disorder (FMD)

#### 3.3.1. Predisposing Factors

Patients frequently exhibited psychiatric comorbidities, especially anxiety disorders, with higher prevalence than in matched neurological controls [2,25].

Personality traits, including dependent, histrionic, and anxious features, may increase sensitivity to bodily sensations and reduce resilience to somatic stress [12]. Evidence of familial modeling was limited but observed in some cases [13].

The role of psychiatric comorbidity was more variable, with associations ranging from 40 to 70% across studies, indicating moderate consistency.

#### 3.3.2. Precipitating Factors

Acute emotional events, particularly relational breakdowns, emotional abuse, or conflict at work, were frequently cited as symptom triggers [25]. In addition, physical pain or injury, including post-surgical states, were described as common precipitating somatic experiences [4].

#### 3.3.3. Perpetuating Factors

Long-term symptom maintenance was often linked to avoidant behavior, such as reduced physical activity or social withdrawal. Patients frequently adopted maladaptive coping strategies, including over-reliance on mobility aids or passive treatments. Negative healthcare encounters, especially those lacking a clear, empathetic diagnostic explanation, were again cited as central contributors to chronicity [8,18].

Table 4 outlines the primary risk factors contributing to functional movement disorder (FMD), categorized in predisposing, precipitating, and perpetuating domains.

### 3.4. Functional Weakness and Sensory Symptoms

A large cohort study by Stone et al. [4] reported that over 60% of patients presenting with motor or sensory functional symptoms identified a proximal trigger, most commonly involving pain, injury, or physical stress.

Long-term maintenance was associated with fear-based avoidance, physical deconditioning, and lack of structured rehabilitation. Importantly, many patients reported that the absence of a coherent, non-threatening diagnostic explanation exacerbated their distress and contributed to prolonged symptomatology [8,19].

### 3.5. Functional Visual Symptoms

In adolescents and young adults, functional visual disturbances were strongly associated with histories of trauma and emotional abuse, particularly in cases involving dissociation or detachment [22]. These symptoms frequently co-occurred with attachment insecurity and fear-driven emotional dysregulation, suggesting a prominent psychogenic component [6].

### 3.6. Functional Gait Disorder

Episodes of functional gait disturbance were frequently triggered by contextual or environmental factors, such as walking in crowded spaces, being observed, or navigating unfamiliar environments [21]. These responses appear to reflect heightened interoceptive sensitivity, anticipatory anxiety, and disrupted motor automaticity, which may reflect broader functional motor system dysregulation.

Table 5 provides an overview of the risk factors reported for functional weakness, sensory disturbances, visual symptoms, and gait disorders, organized according to predisposing, precipitating, and perpetuating categories.

Associations for functional weakness, sensory, visual, and gait symptoms were less frequently studied, and thus evidence strength should be considered preliminary.

### 3.7. Protective and Outcome-Related Factors

A minority of studies reported factors associated with favorable outcomes in FND. These included strong social support, higher levels of insight into the functional nature of symptoms, early and clear diagnostic communication by clinicians, and engagement in multidisciplinary rehabilitation programs. Such protective factors highlight opportunities for improving prognosis and tailoring interventions.

## 4. Discussion

This systematic review synthesized and integrated findings from 23 primary studies, each examining risk factors for Functional Neurological Disorder (FND) through the lens of predisposing, precipitating, and perpetuating mechanisms. The synthesis was conducted across all major FND subtypes and organized using a biopsychosocial framework, offering a structured and comparative interpretation of multifactorial etiological pathways. Although the review protocol followed rigorous systematic review methodology, the study was not registered in PROSPERO due to its advanced stage of completion at the time of review conceptualization, a decision in alignment with PROSPERO’s recommendation to avoid retrospective registration once data extraction has commenced.

This study selection process, detailed in Figure 1 (PRISMA 2020 diagram), allowed the identification of patterns and divergences among FND presentations, with results underscoring the substantial variability and, at times, specificity of contributing factors across subtypes. This level of granularity enriches existing models of FND pathophysiology and suggests potential avenues for personalized formulation and intervention.

### 4.1. Biopsychosocial Risk Architecture Across Subtypes

#### 4.1.1. Predisposing Factors

The most consistently reported predisposing vulnerabilities across all subtypes included histories of childhood trauma (particularly in PNES), psychiatric comorbidities (notably anxiety and depression), personality traits (e.g., harm avoidance, dependency, perfectionism), and neurodevelopmental characteristics (such as ADHD or autism spectrum traits, especially in FCD).

In PNES, childhood trauma emerged as a robust risk factor, supported both by epidemiological data and neurobiological findings indicating altered limbic-prefrontal connectivity in trauma-exposed individuals [6,10]. These results are consistent with trauma models of functional dissociation and stress sensitization, which posit that early adversity predisposes individuals to maladaptive emotional processing and somatic symptom expression under stress.

By contrast, FCD was more strongly associated with neurodevelopmental and metacognitive vulnerabilities. Traits such as perfectionism, attentional dysregulation, and cognitive hypervigilance were frequently noted [14,16,20]. This supports the growing view that FCD lies on a continuum with neurodevelopmental disorders and may arise from internal misattributions of attentional lapses or begin cognitive failures.

Psychiatric comorbidities, especially anxiety, mood disorders, and PTSD, were highly prevalent across all subtypes, but their causal role remains complex. In some cases, psychiatric symptoms predated FND onset, while in others they appeared reactive. This distinction is clinically relevant, as it supports the conceptualization of FND as a heterogeneous disorder where psychiatric burden is an important but not universal contributor [5,15].

Additionally, personality features, although inconsistently operationalized, were more prevalent in FND groups than controls. Traits such as dependency, harm avoidance, or obsessive tendencies may modulate coping under stress or increase vulnerability to symptom misinterpretation [12]. Nevertheless, few studies utilized validated personality inventories, highlighting a gap for future investigations.

#### 4.1.2. Precipitating Factors

More than half of the studies identified acute stressors, physical injuries, or psychosocial upheaval as proximate triggers of symptom onset. These included relational conflict, loss events (e.g., bereavement or job loss), and physical trauma (e.g., surgery, infections, or seizures). Such findings underscore the dynamic interaction between stress response systems and symptom generation in FND [4,6,19].

The review also highlighted the relevance of health system interactions as potential precipitants. Several patients reported onset following adverse medical experiences, ambiguous diagnoses, repeated investigations without answers, or invalidating clinical encounters. These observations align with theories of “iatrogenic suggestion”, where uncertainty and miscommunication may act as nocebo-like stressors, precipitating functional symptoms in vulnerable individuals [8].

FCD and FMD cohorts, in particular, reported a high frequency of physical or psychosocial stressors that temporally preceded symptom development, reinforcing the need to evaluate recent contextual and environmental exposures in clinical assessments.

#### 4.1.3. Perpetuating Factors

Perpetuating factors, those contributing to symptom chronicity, were prominently identified across subtypes and included maladaptive beliefs (e.g., fear of neurological disease), behavioral avoidance, secondary gain (e.g., disability benefits or family accommodation), and inadequate clinician-patient communication.

In PNES and FCD, dysfunctional illness beliefs and catastrophic cognitive styles contributed significantly to symptom persistence. Patients often misinterpreted bodily signals as indicators of severe disease, reinforcing a vicious cycle of fear, monitoring, and further somatic preoccupation [5,20].

Avoidant behavior, especially in FMD and functional weakness, was linked to physical deconditioning, fear-avoidance of movement, and lack of graded exposure to activities [19]. Similarly, social reinforcement, whether from family members, employers, or clinicians, occasionally serves to sustain dysfunctional behavior patterns. These findings mirror cognitive-behavioral formulations and emphasize the role of secondary processes in prolonging symptom expression.

Medical communication emerged as a critical modulator of perpetuation. Across multiple subtypes, patients reported that unclear or stigmatizing explanations increased distress, reinforced functional attributions, and reduced treatment engagement [8]. This supports the recommendation for transparent, empathic, and coherent diagnostic feedback as an essential therapeutic component.

### 4.2. Subtype-Specific Insights and Implications

The findings illustrate both shared and unique risk architectures across FND subtypes:

- PNES: Strongly associated with trauma, psychiatric comorbidity, and dissociative traits. Symptoms often emerge in response to psychosocial crises and are perpetuated by maladaptive coping and social reinforcement.
- FCD: Linked more closely to attentional and metacognitive dysfunctions, perfectionistic traits, and anxiety about cognitive decline. Iatrogenic factors and interpretive ambiguity contribute to symptom maintenance.
- FMD: Personality traits and psychiatric comorbidity appear more salient. Triggers often include physical trauma or psychosocial stress, while perpetuation reflects behavioral avoidance and ineffective communication.
- Functional Weakness/Sensory Symptoms: Typically initiated by minor trauma or pain, with chronicity influenced by disuse, fear-based avoidance, and lack of therapeutic clarity.
- Functional Visual/Gait Symptoms: Characterized by environmental and contextual sensitivity, dissociated traits, and heightened emotional reactivity in public or high-pressure contexts [21,22].

The differentiation has diagnostic and therapeutic value. Understanding that different FND subtypes may reflect dominant pathways (trauma vs. neurodevelopmental vs. behavioral) allows clinicians to tailor formulations and interventions more effectively.

### 4.3. Clinical Land Research Implications

The data reviewed support the utility of individualized biopsychosocial formulations in FND. Rather than a uniform model, risk assessment should incorporate predisposing history (e.g., trauma, neurodevelopmental traits), recent precipitations (e.g., stress, injury), and perpetuating mechanisms (e.g., beliefs, avoidance, systemic influences). This tripartite model may improve diagnostic accuracy, therapeutic alliance, and intervention outcomes.

Taken together, these findings support conceptualizing FND not as a disorder of exclusion or mystery, but as a disorder of integration, in which biological, psychological, and social factors dynamically interact to shape symptom emergence and maintenance.

Importantly, matching interventions to the predominant perpetuating mechanisms has demonstrated efficacy. For example:

- CBT shows benefit in PNES by targeting avoidance and catastrophic misinterpretations [26].
- Multidisciplinary interventions, including physical therapy and psychoeducation, are effective in FMD when they address avoidance and clarify the diagnosis [14].
- Trauma-focused therapies, though underexplored, may be particularly relevant in patients with verified trauma histories [17].

The tripartite structure of predisposing, precipitating, and perpetuating factors can be mapped onto Finkel’s “I^3^ framework” (instigation, impellance, inhibition) [27]. Predisposing vulnerabilities align with impellance, representing stable traits that increase susceptibility to symptoms. Precipitating stressors correspond to instigation, providing immediate triggers for symptom onset. Perpetuating mechanisms reflect deficits in inhibition, where maladaptive beliefs or behaviors prevent recovery. Integrating the review’s findings into this framework may offer a more coherent theoretical model for understanding FND across subtypes.

Future research should prioritize prospective, longitudinal studies to elucidate how clusters of predisposing, precipitating, and perpetuating factors interact over time. Investigating neurobiological and psychophysiological mechanisms underlying these pathways may also guide the development of biomarkers and more targeted interventions.

### 4.4. Strengths and Limitations

This review has several important strengths. It applied a systematic approach to the identification, screening, and synthesis of studies across all major FND subtypes, thereby offering a comprehensive and integrated perspective. The inclusion of both quantitative and qualitative evidence increased the breadth of insights and enhanced the clinical utility of the synthesis. Furthermore, organizing the findings within a biopsychosocial framework provided a coherent structure for interpreting the multifactorial nature of FND.

Nonetheless, several limitations should be acknowledged. Methodological heterogeneity across the included studies, in terms of design, outcome measures, and assessment tools, precluded a formal meta-analysis and limited the comparability of findings. Many studies relied on retrospective designs, introducing potential recall and attribution biases, while only a minority employed validated instruments for key domains such as attachment style, personality traits, or cognitive distortion. As a result, the robustness of some inferences reduces. Variability in study quality and risk of bias also affected the strength of conclusions, with higher-quality studies generally reporting more consistent associations.

Finally, the protocol was not prospectively registered, which reduced procedural transparency; although all steps were predefined and documented, retrospective registration through a platform such as the Open Science Framework (OSF) would have further strengthened methodological rigor.

## 5. Conclusions

Functional Neurological Disorder (FND) arises from a complex interplay of biological, psychological, and social factors rather than a single etiological source. Different FND subtypes, such as psychogenic non-epileptic seizures (PNESs), functional cognitive disorders (FCDs), and functional movement disorders (FMDs), display distinct profiles of risk, emphasizing the need for individualized assessment and treatment.

Predisposing vulnerabilities (e.g., trauma, neurodevelopmental traits, psychiatric comorbidities), precipitating events (e.g., stress, physical injury, psychosocial upheaval), and perpetuating mechanisms (e.g., maladaptive beliefs, avoidance behaviors, and system-level factors) interact dynamically to influence symptom onset and chronicity. Recognizing these mechanisms is essential for accurate diagnosis, personalized case formulation, and effective therapeutic intervention.

Finally, addressing clinician–patient communication, healthcare system interactions, and societal stigma is critical to optimizing engagement in recovery. This review underscores that FND should be conceptualized as a disorder of integration, where multifactorial risk pathways converge, and that tailored, evidence-based, and patient-centered approaches are central to improving outcomes.

## Figures and Tables

**Table 1:** Characteristics of included studies (*n* = 23).

Variable	Summary of Included Studies
Number of studies	23
Publication period	1986–2025
Geographic distribution	Europe, North America, Asia, other
Sample size range	20–300 participants
Study types	Cross-sectional, case-control, cohort, case series
Clinical populations	PNES, FCD, FMD, functional weakness/sensory, visual, gait
Assessment domains	Trauma history, psychiatric comorbidities, personality traits, neuroimaging, neuropsychological testing
Quality appraisal	Majority rated as moderate-to-high quality using standardized tools

**Table 2:** PNES risk factors.

Domain	Key Factors	References
Predisposing	Childhood trauma, anxiety/depressive disorders, personality traits (harm avoidance, emotional lability, cluster B/C traits), familial modeling	[1,2,3,6,7,10,11,12,13]
Precipitating	Acute psychosocial stressors (bereavement, divorce, financial hardship, interpersonal conflict), physical health events (infections, head trauma, seizures)	[4,6,17,18,19]
Perpetuating	Catastrophic illness beliefs, social reinforcement, iatrogenic influence, diagnostic delays	[5,8,18]

**Table 3:** FCD risk factors.

Domain	Key Factors	References
Predisposing	Neurodevelopmental traits (autism spectrum, ADHD), anxiety/depressive disorders, perfectionism, cognitive rigidity	[11,12,13,14,15,20]
Precipitating	Recent stressors, infections, chronic fatigue, sleep disturbances, occupational burnout	[6,13,19]
Perpetuating	Dysfunctional illness models (fear of early dementia), excessive medical testing, poor clinician-patient communication	[5,8,20]

**Table 4:** FMD risk factors.

Domain	Key Factors	References
Predisposing	Anxiety disorders, dependent/histrionic/anxious personality traits, familial modeling	[2,12,13,25]
Precipitating	Acute emotional events, relational breakdown, emotional abuse, pain/injury, post-surgical states	[4,25]
Perpetuating	Avoidant behaviors, maladaptive coping strategies, negative healthcare encounters	[8,18]

**Table 5:** Other functional symptoms (weakness, sensory, visual, gait).

Domain	Key Factors	References
Predisposing	Trauma history, emotional abuse, attachment insecurity, heightened interoceptive sensitivity	[6,22]
Precipitating	Pain, injury, physical stress, contextual/environmental triggers (crowded spaces, observation)	[4,19,21]
Perpetuating	Fear-based avoidance, physical deconditioning, diagnostic uncertainty, disrupted motor automaticity	[8,19]

## Data Availability

The data presented in this study are included in the article. Additional data are available from the corresponding author on request.
